# Associations between Parental Concerns about Preschoolers’ Weight and Eating and Parental Feeding Practices: Results from Analyses of the Child Eating Behavior Questionnaire, the Child Feeding Questionnaire, and the Lifestyle Behavior Checklist

**DOI:** 10.1371/journal.pone.0147257

**Published:** 2016-01-22

**Authors:** Anna Ek, Kimmo Sorjonen, Karin Eli, Louise Lindberg, Jonna Nyman, Claude Marcus, Paulina Nowicka

**Affiliations:** 1 Division of Pediatrics, Department of Clinical Science, Intervention and Technology, Karolinska Institutet, Stockholm, Sweden; 2 Division of Psychology, Department of Clinical Neuroscience, Karolinska Institutet, Solna, Sweden; 3 Unit for Biocultural Variation and Obesity, Institute of Social and Cultural Anthropology, University of Oxford, Oxford, United Kingdom; 4 Department of Food, Nutrition and Dietetics, Uppsala University, Uppsala, Sweden; TaipeiCityHospital, TAIWAN

## Abstract

**Introduction:**

Insight into parents’ perceptions of their children’s eating behaviors is crucial for the development of successful childhood obesity programs. However, links between children’s eating behaviors and parental feeding practices and concerns have yet to be established. This study aims to examine associations between parental perceptions of preschoolers’ eating behaviors and parental feeding practices. First, it tests the original 8-factor structure of the Child Eating Behavior Questionnaire (CEBQ). Second, it examines the associations with parental feeding practices, measured with the Child Feeding Questionnaire (CFQ).

**Materials and Methods:**

Questionnaires were sent to parents from 25 schools/preschools in Stockholm, Sweden and to parents starting a childhood obesity intervention. The CEBQ factor structure was tested with confirmatory factor analysis (CFA). Associations between CEBQ subscales Food approach and Food avoidance and CFQ factors Restriction, Pressure to eat and Monitoring were examined with structural equation modelling (SEM), adjusting for child and parental characteristics, and parental confidence, measured with the Lifestyle Behavior Checklist (LBC). CFQ Concern for child weight and Perceived responsibility for child eating were used as mediators.

**Results:**

478 parents completed the questionnaires (children: 52% girls, mean age 5.5 years, 20% overweight/obese). A modified 8-factor structure showed an acceptable fit (TLI = 0.91, CFI = 0.92, RMSEA = 0.05 and SRMR = 0.06) after dropping one item and allowing three pairs of error terms to correlate. The SEM model demonstrated that Food approach had a weak direct effect on Restriction, but a moderate (β = 0.30) indirect effect via Concern, resulting in a substantial total effect (β = 0.37). Food avoidance had a strong positive effect on Pressure to eat (β = 0.71).

**Discussion:**

The CEBQ is a valid instrument for assessing parental perceptions of preschoolers’ eating behaviors. Parental pressure to eat was strongly associated with children’s food avoidance. Parental restriction, however, was more strongly associated with parents’ concerns about their children’s weights than with children’s food approach. This suggests that childhood obesity interventions should address parents’ perceptions of healthy weight alongside perceptions of healthy eating.

## Introduction

Parents’ responses to children’s appetitive traits may have profound consequences for the development of children’s food preferences, eating habits, and body weights [1–7]. Links between child eating behavior and parental feeding practices, therefore, have been the focus of several research studies [1, 8–10]; however, the clinical implications of these links remain unclear. This study aims to examine associations between parental perceptions of preschoolers’ eating behaviors and parental feeding practices. Identifying these associations is important for the development of a clearer framework to guide childhood obesity interventions—a framework that accounts for the co-constitutive dynamics of eating and feeding in the family context.

It is particularly important to study parental feeding practices and children’s eating behaviors during the preschool age: children are still highly dependent on their parents for the structuring of food intake, but are also developing more autonomy through preschool and social interactions with other children, under the supervision of other adults and outside their parents' direct control. The communication between children and their parents becomes more developed than in earlier ages [11–13]. With infants and toddlers, parents can make all feeding decisions and respond to the child with a simple yes or no; responding to a preschooler’s food demands, however, is more complex. The preschool age is therefore a crucial time in which parents develop communication about food with their children [1]; indeed, previous studies have shown that obesity interventions may be most effective in the preschool age range [14, 15].

The preschool age is a time when distinct eating behaviors are formed [16]. Twin and family studies have suggested that there are genetic predispositions for some obesity related appetitive traits such as food preferences [17], speed of eating [18], eating in the absent of hunger (for boys) [19, 20], satiety responsiveness and food responsiveness [21]. However, children’s eating behaviors are also significantly influenced by social and environmental factors such as role modeling [22, 23], availability of food in the home [24, 25] and parenting practices [1, 26–28]. Thus, understanding how and why parents respond to children’s different eating behaviors is key to framing childhood obesity interventions.

The tool used most commonly to describe children’s eating behaviors is the Child Eating Behavior Questionnaire (CEBQ) [29–31]. The CEBQ includes a list of statements that characterize children’s appetites, clustering responses into eight factors divided into two main dimensions: Food approach (factors: Food responsiveness, Emotional overeating, Enjoyment of food and Desire to drink) and Food avoidance (factors: Satiety responsiveness, Slowness in eating, Emotional undereating and Food fussiness) [30, 31]. High scores on Food approach have been associated with higher weight status while high scores on Food avoidance have been associated with a lower weight status among preschoolers [32–34], school aged children [32, 34–36] and adolescents [34, 37].

Among obesity related parenting practices, three feeding behaviors have been studied most often: restriction (the extent to which parents control the child’s consumption of sugary and high-fat food), pressure to eat (the extent to which parents encourage the child to eat) and monitoring (the extent to which parents direct the child toward healthy eating) [1]. A number of studies, predominantly of a cross sectional design, have found positive associations between parental restriction, food approach, and child weight status [9, 35, 38, 39]. Likewise, pressure to eat has been positively associated to Food avoidance as well as with lower child weight status [33, 38]. Longitudinal studies have confirmed these relationships, suggesting that parents seem to adapt their feeding practices to child behavior and child weight [25, 40, 41]. In addition, several studies have examined parental cognitions, such as concern for child weight, as mediators for feeding practices (e.g., restrictive or pressuring feeding practices increase only when parents become concerned about their children’s weights) [38, 42–44]. This is particularly notable because parental recognition of and concern about child overweight is required for successful recruitment to obesity treatment studies [45]. In preschool age, however, parents and other adult family members find it difficult to identify child weight status correctly [46, 47]. Still, the relationship between parental concern about children’s weight and parents’ ability to make the best feeding decisions for their children is not straightforward. High levels of parental restrictive feeding practices can be counterproductive. In longitudinal studies, children developed an increased preference for the foods their parents restricted [27, 48, 49]; the opposite occurred when parents pressured children to eat foods they resisted [50]. In effective intervention studies, both parental concern and restrictive parenting practices diminished over time [51–53]. To sum up the above findings, there seem to be a paradox: some level of parental concern is needed for parents to reflect on and change their feeding behaviors; however, too much concern may lead to counterproductive feeding practices.

Parental concern, then, is a complex factor that needs to be further investigated. Differences in the extent and expression of parental concern may reflect cultural background, child and parental gender, age and socio-economic status; the same factors may influence the use of restrictive, monitoring, or pressuring feeding practices [54–60]. In a Swedish population based study, parents of preschoolers reported lower levels of restrictive feeding practices than parents in Australia, USA and Japan [58]. Other studies have examined the higher levels of parental concern about child weight and the use of more restrictive feeding practices among parents of girls [59, 61]. Parental self-efficacy (also referred to as confidence) is another factor to consider as a predictor of parenting practices [62] in relation to a child’s healthy lifestyle [63]. In Australia, parents’ confidence in their handling of children’s problematic weight related behaviors, measured with the Confidence scale of the Lifestyle Behavior Checklist (LBC) [64, 65], increased after a parent-centered obesity intervention [66]. In the validation of the Swedish LBC, we found significant associations between parental confidence and parental concern, restriction and pressure to eat [67]. In the present study we therefore include parental confidence in our model as a predictor of parenting practices. Likewise, understanding the underlying factors (other than weight status) for child eating behaviors is of great importance, in clinical practice as well as in research; however, data are still inconclusive about differences regarding gender [32, 36, 68, 69], age [16, 31, 32, 36, 69] and cultural background [70–72].

The overall aim of this study was to present a comprehensive model of associations between parental perceptions of child eating behaviors among preschoolers and parental feeding practices, adjusting for potentially important predictors. The first aim was to establish the psychometric properties of a Swedish version of the CEBQ in preschoolers [69], examining the original 8-factor structure using confirmatory factor analysis (CFA)–an analysis that has not been done before. The second aim was to test a model of the direct and indirect effects of the two CEBQ dimensions (Food approach and Food avoidance), as well as child and parental characteristics on the Child Feeding Questionnaire (CFQ) factors Restriction, Pressure to eat and Monitoring. We focus on parents of children between the ages of 3 and 8 years. As Swedish children attend preschool until the age of 7 years, we refer to the children as preschoolers throughout the paper.

Based on previous research [33, 40], we created a model hypothesizing that parental feeding practices are affected by parents’ perceptions of child eating behaviors and that parents’ concerns about child weight would mediate this relationship [38, 43]. We controlled for child and parental characteristics as the previous research findings have been inconclusive. However, we hypothesize that two of these will be most important: 1) child weight status, because of its association with eating behaviors [33, 35, 73] and feeding practices [1, 39], and 2) parental education, a common proxy for socioeconomic status, due to its associations with obesity [74].

## Materials and Methods

### Study participants

The recruitment process has been described comprehensively elsewhere [75]. In summary, to obtain a representative sample of preschoolers with a variation in weight status, the researchers contacted preschools and schools in different areas of Stockholm County. The selected preschools and schools represented areas with low, medium or high prevalence of obesity, as recorded in data from the most recent primary care report [76]. Forty-five institutions were contacted (30 preschools and 15 schools); 25 agreed to participate (20 preschools and 5 schools). A total of 931 parents received the CEBQ, CFQ, LBC and a background questionnaire: 595 parents with children attending preschool and 336 parents with children in the preparation year of school. Participants sent the questionnaires back to the research group in an enclosed envelope. All data were collected anonymously. We also added baseline data from a clinical population of parents (n = 47) of preschoolers with obesity, who were referred by primary child care centers in Stockholm County to a randomized controlled childhood obesity trial (NCT01792531). Written informed consent was obtained from all participants. The caretakers provided written informed consent on behalf of the children enrolled in the study. The ethics committee approved the consent procedure. Both the validation study and the clinical study were approved by the Regional Ethics Board in Stockholm (dnr: 2011/1329-31/4, 2012/1104-32, 2012/2005-32, 2013/486-32, 2013/1628-31/2).

### Study instruments

#### The CEBQ

The CEBQ includes 35 items on eating styles related to obesity risk, which cluster into eight factors [31]. The ‘Food approach’ dimension is represented by four factors: Food responsiveness, with five items (e.g. “given the choice, my child would eat most of the time”), Emotional overeating, with four items (e.g. “my child eats more when worried”), Enjoyment of food, with four items (e.g. “my child enjoys eating”), Desire to drink, with three items (e.g. “my child is always asking for a drink”). The ‘Food avoidance’ dimension is represented by: Satiety responsiveness, with five items (e.g. “my child gets full up easily”), Slowness in eating, with four items (e.g. “my child finishes his/her meal quickly”), Emotional undereating, with four items (e.g. “my child eats less when upset”), and Food fussiness, with six items (e.g. “my child refuses new foods at first”). Parents rate each behavior on a five-point Likert scale (never, rarely, sometimes, often, always; 1–5). The CEBQ was proven to have a good validity and high internal reliability in the United Kingdom, where it was developed (among parents of 2-9-year-olds) [30, 31], and, following some adaptations, in Portugal (3-13-year-olds) [34], the Netherlands (6-7-year-olds) [32], Canada (4-5-year-olds) [77], Chile (6-12-year-olds) [36], China (1–1.5-year-olds) [68], Australia (1–5 year olds) [78], Malaysia (13-year-olds) [72], the United States (2–5 year-olds) [79] and Sweden (1–6 year-olds) [69]. However, CFA, the gold standard for validating questionnaires which have already been developed, has been used only in the U.S. [79] and in the Australian validation studies [78]. The Swedish validation of the CEBQ version used in the present study has been tested with exploratory factor analysis [69], which is more data- than theory-driven. In that validation study, the established 8-factor structure was not replicated, possibly due to the small sample size. Instead, the study proposed a 7-factor structure, in which the factors Food responsiveness and Emotional overeating were combined into one factor.

#### The CFQ

The CFQ evaluates parents' concerns about their children’s body weights, and their child-feeding practices [80]. The CFQ is comprised of seven factors. Four factors measure parental perceptions of body weight, both their child’s and their own, and concerns that may affect parental control of children’s eating: Perceived responsibility, with three items (e.g. “When your child is at home, how often are you responsible for feeding her?”); Perceived parent weight, with four items (e.g. during the participant’s “childhood [5 to 10 years old]”); Perceived child weight, with three items (e.g. “during the first year of life”); and Concern about child weight, with three items (e.g. “How concerned are you about your child becoming overweight?”). Three factors measure parents’ feeding practices, including: Restriction, with eight items (e.g. “I intentionally keep some foods out of my child’s reach”); Pressure to eat, with four items (e.g. “My child should always eat all of the food on her plate”); and Monitoring, with three items (e.g. “How much do you keep track of the high fat foods that your child eats?”) [80]. The Swedish version of the CFQ was used in this study. This version was validated in a recent population-based study, which involved parents of preschoolers [58].

#### The LBC

The LBC consists of 25 items, divided into two scales: the Problem scale and the Confidence scale [64, 65]. The Problem scale assesses parents’ perceptions of children’s obesity-related problem behaviors. The Confidence scale assesses how confident parents feel about handling their children’s problematic obesity-related behaviors. The original four factor structure included; Misbehavior in relation to food, with seven items (e.g. the child yells about food), Overeating, with seven items (e.g. the child eats too much), Emotional correlates related to being overweight, with five items (e.g. the child complains about being overweight) and Physical activity, with five items (e.g. the child complains about being physically active). In the Swedish version of the LBC, validated in the same population of parents as the present study, the CFA suggested a five factor structure where the Physical activity factor was divided into an additional factor, Screen time (e.g. child watches too much TV) [67]. Both scales use Likert ratings: on the Problem scale, parents rate the extent to which they perceive a child’s behavior as problematic, from 1 (“not at all”) to 7 (“very much”); on the Confidence scale, parents rate the extent to which they feel they can handle a problematic behavior, from 1 (“certain I can’t do it”) to 10 (“certain I can do it”). Parents who have not encountered the problematic behaviors the instrument lists are asked to estimate how confident they would feel if these situations occur. The LBC has shown high internal reliability and good consistency with other instruments measuring child behavior and parenting [29, 64, 65, 67]. In the validation of the LBC, the CFA indicated that the Confidence scale was unidimensional and was not associated with any child or parental characteristics [67]. Together, these results indicate that confidence might be an independent factor, i.e. parents either have or do not have the confidence to handle problematic lifestyle-related child behaviors. In the present study, we therefore use confidence as a predictor of parental feeding practices.

### Statistical analysis

The descriptive statistics are presented as means and standard deviations (SD), or numbers and percentages for categorical data. Independent two-tailed t-tests (for continuous variables) and chi square tests (for categorical variables) were used to report the differences between the school sample and the clinical sample. All p-values < 0.05 were regarded as statistically significant. These analyses and the reliability calculations (Cronbach’s alpha) were conducted with SPSS version 22. MPlus version 7.11 was used to perform CFA and structural equation modelling (SEM), using full information maximum likelihood (FIML) with robust standard errors estimation, which allows for missing data. Even when the missing at random (MAR) assumption is not met, FIML produces less biased estimates than listwise deletion. CFA is recommended to test factorial validity when previous hypotheses about the dimensions of the construct are available based on theory and/or previous analysis [81].The original eight-factor model of the CEBQ [30] was tested with CFA. To examine fit to the data, four commonly recommended fit indices were used: the comparative fit index (CFI), the Tucker-Lewis Index (TLI), the root mean square error of approximation (RMSEA) and standardized root mean square residual (SRMR). Adequate fit was indicated by CFI and TLI values over 0.90 [82] and good fit was indicate by values over 0.95, a RMSEA of 0.06 or lower and a SRMR of 0.08 or lower [83]. By analyzing associations between latent variables with SEM, rather than associations between observed variables with ordinary regression, we allow measurement error in independent, as well as dependent, variables [84]. This tends to increase the power of the analyses. Moreover, SEM is very useful when analyzing mediated effects.

SEM was performed where five CFQ factors (Restriction, Pressure to eat, Monitoring, Perceived responsibility and Concern for child weight) were regressed on the CEBQ subscales Food approach and Food avoidance and the background variables, which include three child characteristics (gender, age, body mass index standard deviation score (BMI SDS) and five parental characteristics (gender, age, BMI, foreign origin, and education level), as well as on the LBC Confidence scale. Concern for child weight and Perceived responsibility were used as mediators in the model. The CFQ factors Perceived parent weight and Perceived child weight were not included as predictors as they contributed little unique variance (tolerance < 0.4). Effect sizes and correlations are assessed according to Cohen (0.1–0.3 weak; 0.3–0.5 medium, 0.5–1 strong) [85].

Child weight categories were created using age and gender specific international cut offs for BMI [86]. Child BMI SDS was derived using Swedish age and gender specific reference values [87].

### Terminology

Throughout this paper, the term 'predictor' is used as a statistical term and not as an indication of causality, considering that we have cross-sectional data. Regarding the use of the term ‘influence/influential’: we are aware of Reichenbach’s principle [88], and therefore we do not claim to determine causality. Our use of the words 'predictor', 'influence' and 'effect on' reflects our definition of some factors as independent and others as dependent in relation to each other in the models.

## Results

### Descriptive statistics

In total 432 questionnaires were returned to the research group (one questionnaire had incomplete answers, and was therefore excluded from the analysis). Child and parental characteristics are presented in Table 1 and have been described comprehensively elsewhere [67]. In summary, in the total sample of parents (n = 478) (mean age 38.9, SD = 5.0), 81% were women, 70% had a university degree, 87% had Nordic background, 31% were overweight/obese (based on self-reported weight and height). In the clinical sample (n = 47), the parents had lower education and higher levels of overweight and obesity, and higher percentages were born outside Sweden. Among the children (mean age 5.5, SD = 1.0, range 3.3 to 7.95 years), 52% were girls, 20% with overweight/obesity.

**Table 1:** Characteristics of the samples.

Variable	Total population (n = 478)		Clinical sample (n = 47)		School sample (n = 431)	
**Continuous**	**Mean**	**SD**	**Mean**	**SD**	**Mean**	**SD**
Child’s age (years)	5.5	1.0	5.1	0.7	5.5	1.0
Parent’s age (years)	38.9	5.0	37.6	7.2	39.0	4.7
Child BMI SDS	0.2	1.4	3.1	0.7	-0.2	0.95
Mother BMI	23.6	3.9	27.6	5.8	23.3	3.3
Father BMI	25.5	2.9	26.9	3.7	25.3	2.8
**Categorical**	**n**	**%**	**n**	**%**	**n**	**%**
*Child gender*						
Female	249	52	25	53	224	52
Male	227	48	22	49	205	48
*Parent gender*						
Female	388	81	37	79	351	81
Male	90	19	10	21	80	19
*Country of origin*						
Nordic	411	87	26	55	385	90
Non-Nordic	64	13	21	45	43	10
*Mother’s education*						
University degree	274	71	17	46	257	74
No university degree	111	29	20	54	91	26
*Father’s education*						
University degree	58	65	6	60	52	66
No university degree	31	35	4	40	27	34

### Factorial validity and internal reliability of the CEBQ

**Fig 1:**
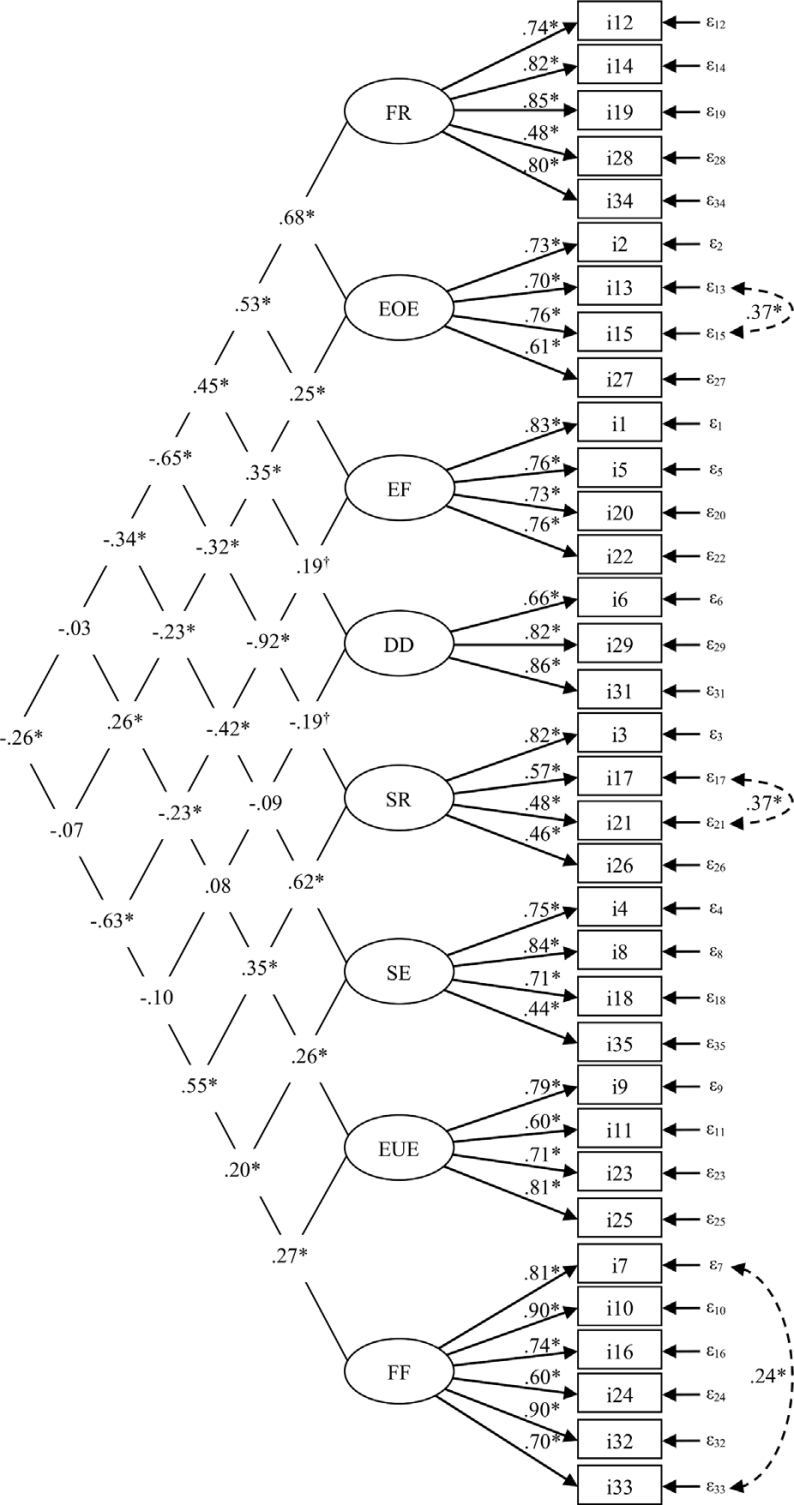
A Confirmatory Factor Analysis of the Child Eating Behavior Questionnaire (CEBQ). The CEBQ eight factors: Food Responsiveness (FR), Emotional Overeating (EOE), Enjoyment of Food (EF), Desire to Drink (DD), Satiety Responsiveness (SR), Slowness in Eating (SE), Emotional Undereating (EUE) and Food Fussiness (FF). Item 30 was dropped as it had a factor loading < 0.4 and three pairs of error terms were allowed to correlate. The model shows acceptable fit to data, χ^2^(496) = 1102, *p* < 0 .001; TLI = 0.91; CFI = 0.92; RMSEA = 0.05 (90% CI: 0.047–0.055) and SRMR = 0.06). ^†^ *p* < 0.05; * *p* < 0.001. The estimates on the left side in the figure stand for correlations between the factors and the estimates on the right side of the figure stand for factor loadings.

The fit of the original 8-factor structure as well as the 7-factor structure proposed in a previous Swedish validation [69] was poor. Instead, CFA demonstrated an acceptable fit (TLI = 0.91, CFI = 0.92, RMSEA = 0.05 and SRMR = 0.06) for a modified 8-factor structure after dropping item 30 on the Satiety responsiveness factor (“my child cannot eat a meal if s/he has had a snack just before”) as it had a loading of < 0.40. After consulting modification indices, three pairs of error terms were allowed to correlate as this resulted in a substantial improvement in model fit. However, only correlations within factors were allowed. These were item 13 (“eats more when annoyed”) and 15 (“eats more when anxious”) on Emotional overeating, 17 (“leaves food on his/her plate at the end of a meal”) and 21 (“gets full before his/her meal is finished”) on Satiety responsiveness, and finally 7 (“refuses new foods at first”) and 33 (“decides that s/he doesn’t like a food, even without tasting it”) on Food fussiness. The correlation between the factors Enjoyment of food and Satiety responsiveness was extremely strong (-0.92). Enjoyment of food also correlated strongly with Food fussiness (-0.63). Food responsiveness correlated strongly with Emotional overeating (0.68) and Satiety responsiveness (-0.65). The CFA is presented in Fig 1.

The internal consistency was adequate (Cronbach’s alphas above 0.7) for all factors. Unweighted mean factor scores (± SD) and internal reliability estimates (Cronbach’s alphas) for CEBQ factors are presented in Table 2 for descriptive purposes.

**Table 2:** Descriptive statistics and Cronbach’s alphas for each factor.

Child Eating Behavior Questionnaire Factors	Mean (SD)	Cronbach’s alpha
Food responsiveness, 5 items	1.73 (0.71)	0.83
Emotional overeating, 4 items	1.40 (0.51)	0.75
Enjoyment of food, 4 items	3.40 (0.69)	0.85
Desire to drink, 3 items	1.83 (0.76)	0.81
Satiety responsivness, 4 items*	3.16 (0.66)	0.74
Slowness in eating, 4 items	2.83 (0.82)	0.77
Emotional undereating, 4 items	2.85 (0.82)	0.81
Food fussiness, 6 items	2.66 (0.87)	0.90

Behaviors are rated on a five-point Likert scale (1 = never, 2 = rarely, 3 = sometimes, 4 = often, 5 = always).

* Not including one item from the original scale “My child cannot eat a meal if she has had a snack just before” that was excluded after performing confirmatory factor analysis.

### Child eating behaviors and parental feeding practices and concerns

**Fig 2:**
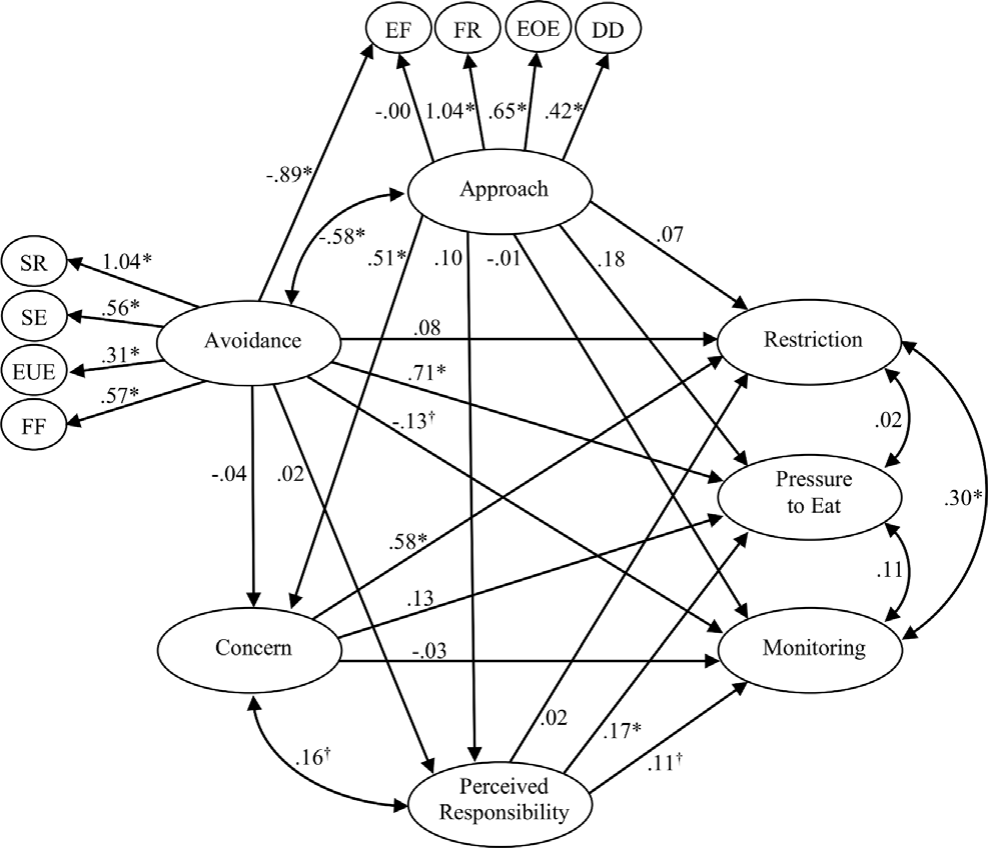
A structural equation model of child eating behaviors and parental feeding practices. The model shows associations between child Food approach and Food avoidance (measured with the Child Eating Behavior Questionnaire) and parental Restriction, Pressure to eat, and Monitoring, with Concern and Perceived responsibility (measured with the Child Feeding Questionnaire) as mediators. The effects (standardized) are adjusted for the effects of child age, gender and body mass index standard deviation score and for parental age, gender, body mass index, foreign origin (Nordic/non-Nordic) and education level (University degree or not) as well as for parental life-style specific Confidence (measured with the Lifestyle Behavior Checklist’s Confidence scale). The model shows mediocre fit to data, χ^2^(2718) = 5072, *p* < 0.001; TLI = 0.878; CFI = 0.885; RMSEA = 0.043 (90% CI: 0.041–0.044); SRMR = 0.07. ^†^ *p* < 0.05; * *p* < 0.001.

Fig 2 shows a model with CEBQ Food approach and Food avoidance and their effects on parental Restriction, Pressure to eat, and Monitoring through Concern and Perceived responsibility. Several child and parental characteristics were used as predictors; for simplicity, they are not visualized in the Figure, but provided in Table 3. The results show a strong positive direct effect of Food avoidance on Pressure to eat (β = 0.71; p < 0.001). Food approach did not have any strong or significant direct effects on parental feeding behaviors (Restriction β = 0.07; Pressure to eat β = 0.18; Monitoring β = -0.1); however, it had a moderate (β = 0.30) indirect effect on Restriction via Concern, which resulted in a substantial total effect (β = 0.37). The independent predictive effect of Food approach on parental Concern was strong (β = 0.51, p < 0.001) as well as the direct effect of Concern on parental Restriction (β = 0.58, p < 0.001).

**Table 3:** Correlations between purely exogenous (independent) variables in the model and their standardized effects on endogenous (dependent) variables.

	1.	2.	3.	4.	5.	6.	7.	8.	9.	10.	11.
*Child*											
1. Food approach	-	-.583*	-.029	.098^†^	.579*	-.092	-.010	-.234*	-.155^†^	.210*	-.205*
2. Food avoidance		-	-.008	-.032	-.396*	.059	-.028	.082	.053	-.021	.017
3. Age			-	-.011	-.030	.156*	-.028	.090^†^	.006	-.068	.074
4. Girl				-	.105^†^	-.093^†^	-.054	-.041	-.096^†^	.015	-.040
5. BMI SDS					-	-.055	-.045	-.209*	-.157^†^	.303*	-.104^†^
*Parental*											
6. Age						-	-.170*	.062	.126^†^	.024	.014
7. Woman							-	.004	.109^†^	-.187*	.026
8. Nordic								-	.133^†^	-.136^†^	.108
9. University									-	-.136^†^	-.046
10. BMI										-	-.072
11. Confidence											-
*Effect on*											
Concern	.514*	-.039	-.021	.017	.331*	-.053	.020	-.157*	.016	.105*	-.010
Perceived Resp.	.099	.021	.080	.029	-.024	-.095	.221*	-.170^†^	-.148^†^	.038	.047
Restriction	.072	.077	-.042	.105^†^	.022	.015	-.030	-.084	.029	.037	-.175*
Pressure eat	.183	.712*	-.003	.022	-.220^†^	-.077	-.043	-.041	.012	.028	-.121^†^
Monitoring	-.012	-.133^†^	-.040	.057	.062	-.023	-.035	.173^†^	.010	-.029	.025

^†^ *p* < 0.05

* *p* < 0.001

The effect of each variable 1–11 in this table on Concern, Perceived responsibility, Restriction, Pressure to eat, and Monitoring is adjusted for the effects of all the other variables 1–11.

University: A university degree.

Confidence: Parental confidence in handling child problematic behavior related to obesity.

BMI: Body Mass Index.

BMI SDS: Body Mass Index Standard Deviation Score.

### Associations of child and parental characteristics

The correlations between the eleven predictors are presented in Table 3. Excluding the correlation between Food approach and Food avoidance, Food approach had its strongest correlation with child’s weight status (BMI SDS) (r = 0.58, p < 0.001). Parental Nordic origin, education level, BMI and Confidence were all only weakly (r < 0.3) associated with Food approach, although the correlations were significant. Other than a significant correlation with Food approach, Food avoidance was significantly correlated only with child’s weight status (BMI SDS) (r = -0.40, p < 0.001). Child BMI SDS also had a moderate independent predictive effect on parental Concern (β = 0.33, p < 0.001). Beside the child’s BMI SDS, parental sociodemographic factors had significant influence on Concern and Perceived responsibility; the effects, however, were also weak

## Discussion

### Summary of the main findings

The study demonstrates that the Swedish version of the CEBQ is a valid instrument for assessing parental perceptions of preschoolers’ eating behaviors. The analysis of links between child eating behaviors and parental feeding practices reveals two important findings. First, parents who are concerned about their child’s weight are more likely to report high levels of restrictive feeding practices, compared to parents who only perceive their child as having a big appetite, and are not concerned about the child’s weight. However, the results suggest that parents who identify children as picky eaters are more likely to report high levels of pressure to eat. Second, among predictors, child BMI SDS was the only variable that was moderately and significantly associated with both dimensions of child eating behaviors (Food approach (positive association) and Food avoidance (negative association)). Child BMI SDS also significantly predicted parental concern for child weight. Together, the findings suggest that parental perception of children’s small appetites is closely associated to the use of pressure to eat, while parental concern about children’s overweight is closely associated with restrictive feeding practices.

### Validity of the CEBQ

Previous factorial validations of the CEBQ have indicated problems at both the item and the factor levels when the questionnaire was adapted to new populations [34, 36, 68, 72, 78]. Compared to the CEBQ’s other international adaptations, the structure proposed in this study is very similar to the original structure, and only the Australian version is as similar [78]. This may be due to the fact that both this study and the Australian study used CFA, which is the appropriate method for such validation, and both had large sample sizes. The snacking item (“My child cannot eat a meal if s/he has had a snack just before”) that loaded weakly on Satiety responsiveness and was excluded in this study, was also problematic in the Australian study [78]. A recent U.S. based study on mothers’ perceptions of feeding snacks to their preschoolers found that mothers define snacking variably: some mothers count all eating occasions as snacking, while others classify snacks as distinct from “real food” [89], for nutritional and other reasons. In our validation of the LBC, we also experienced problems with an item on snacking (“Eats unhealthy snacks”) [67]. Thus, we suggest that Swedish parents may also find it difficult to define snacking, as snacks may range from an apple or an ice-cream to a small organized meal in preschool or at home. Another issue, which is more specific to Sweden, is that most young children spend a major part of the day in kindergarten or preschool with a structured meal schedule, such that parents might not be sure about what the child has eaten during several hours of the day. Therefore, the snack measure to define satiety responsiveness may be difficult for parents to address.

### Child eating behaviors and parental feeding practices

Our results extend the findings from previous research that indicated that parental feeding behavior is closely associated with child eating behaviors [25, 33, 55, 73, 90–93] and parental concern for child weight [38, 42], but not necessarily with the child’s actual weight [38]. Our model shows that parents who perceive their children as having a small appetite are more likely to report exerting pressure to eat. It should be noted, however, that parental perceptions of a small appetite do not necessarily mean that the child is eating too little. Perceptions of a small appetite may reflect parents’ difficulties with assessing appropriate portion sizes and recommended intake for preschoolers, or their lack of trust in the child’s ability to self-regulate food intake [7]. It is therefore important to inform parents about portion sizes and children’s hunger and satiety ques as part of childhood obesity prevention and intervention programs. As previously shown [38], our model did not demonstrate a strong association between children’s Food approach and parental feeding practices. In the preschool age group, parents may be more attuned to children’s undereating, and it is not until parents are concerned for the child’s weight they change their feeding practices. This is complicated by the fact that parents find it hard to identify their preschool aged child’s weight correctly [94]. The results point to the importance of educating clinicians in communicating child weight status to parents [45], in order to support effective feeding practices and to avoid ineffective practices.

The weak association between Food approach and Restriction in this sample could be explained by the items representing the Restriction factor as well as child age. The items “I have to be sure that my child does not eat too many sweets (candy, ice cream, cake or pastries)”, “I have to be sure that my child does not eat too many high-fat foods”, “I intentionally keep some foods out of my child's reach”, “If I did not guide or regulate my child's eating, she would eat too much junk foods” can be applicable to most preschoolers, with parents wanting to limit sweets, fatty foods and junk foods regardless of appetite. Further, in this age group, most of the food children eat is provided by their parents; if the parents do not provide certain foods (e.g., candy), they do not have to restrict these foods, even if a child has a big appetite [95]. In our model, we excluded the two reward items from the CFQ Restriction factor (“I offer sweets (candy, ice cream, cake, pastries) to my child as a reward for good behavior” and “I offer my child her favorite foods in exchange for good behavior”), which may have contributed to a lower effect of Food approach on Restriction. In the validation study of the CFQ among Swedish parents, we discussed how these questions, although relevant, are problematic due to social desirability: parents know that food (especially sweets and snacks) as reward should be avoided, and are likely to respond to these items according to social norms [58].

There was a weak, but significant, negative association between Food avoidance and Monitoring. Monitoring was also weakly associated with Perceived responsibility. However, neither parental Concern nor Perceived responsibility mediated the effects of child eating behavior on parental monitoring behavior. These findings are not surprising, as previous studies also did not find strong associations between child eating behavior and parental monitoring: among the parents of Australian preschoolers, no association was found between parental monitoring and children’s eating in the absence of hunger [10], food fussiness and food responsiveness [38]. Similar to the role modelling of healthy eating [38], monitoring may reflect the food environment the parent offers the child and is not a response to the child’s eating [1, 38]. This may explain associations between monitoring and healthier child weight development [40], as well as why parents do not apply different monitoring practices to siblings of differing weight statuses [96] or eating behaviors [97].

### Parental cognitions

Concern for child weight has been identified previously as increasing parental restrictive feeding [42–44, 59]. In this sample, Concern was not only strongly associated with a parent’s perceptions of the child’s high level of Food approach, but also mediated most of the association between children’s Food approach and parental restrictive feeding practices. We explored the mediating role of Concern using a theory driven statistical approach, including all scales of the CEBQ and adjusting the model for a wide range of child and parental characteristics. Concern as a motivator for parental behavior is important for professionals to recognize and to understand. Longitudinal data on American 7–14 year-olds showed that a higher level of maternal concern was associated with a slower increase in child fat mass [60]. Examining results from obesity interventions, Burrows and colleagues reported a decrease in concern about child weight after 6 months; however, concern returned to baseline levels after 12 months [53]. The results indicate that during the intervention, when parents receive clinical support in managing the child’s weight, concern decreases, but when clinical support ends, concern may increase again. Identification of factors that drive parental concern about child weight, such as socially influenced body weight ideals and knowledge about the health consequences of obesity, that are not explicitly studied in connection to concern about child weight [80], can help professionals provide parents with appropriate information and coping strategies. However, it is important to acknowledge that we did not collect data on parental concern about child underweight; this likely explains why concern for child weight did not mediate effects on parental Pressure to eat, as has been reported previously [38].

Parental confidence in handling child eating behaviors related to obesity [64, 65] was used as a predictor of parental feeding practices in the model. In a validation of the LBC [67], the Confidence scale proved to be unidimensional, indicating that confidence is a global construct, not specific to certain situations or behaviors. We only detected weak negative associations, although significant, between parental Confidence and Restriction and Pressure to eat. The negative association is logical, since a high level of Restriction or Pressure to eat indicates lower Confidence in handling problematic child eating behaviors, such as a too large or too small appetite.

### Sociodemographic predictors

Awareness of determinants and predictors of child eating behaviors and parenting practices helps to develop successful childhood obesity interventions. We examined the effects of several child and parental characteristics: age, gender, weight status, parental foreign background and parental education level. However, only child weight status (BMI SDS) had a moderate to strong significant association with child eating behavior and parental Concern. Child weight has been identified previously as a predictor for child eating behavior: a lower weight status was predictive of Food avoidance and a higher weight status of Food approach [27, 32, 36].

We found a significant independent association between child BMI SDS and parental concern about child weight, but, like Gregory et al. [38], child weight status was not an independent predictor for parental restrictive feeding practices. However, we did observe a significant, though weak, negative association between BMI SDS on Pressure to eat. Parents may respond to a child’s perceived weight, but this perceived weight does not always correspond to the child’s actual weight [38]. We used weight status as a continuous variable and did not divide children into different weight categories, hence we cannot draw conclusions about whether parental concern reflected children’s actual weight status (e.g. if the child was underweight, normal weight, overweight or obese). Beside the child’s BMI SDS, parental sociodemographic factors were significantly associated to Concern and Perceived responsibility; the associations, however, were also weak. This means that although some parental characteristics are associated with their feeding practices, child characteristics–in particular, perceived weight status–may influence parents more.

### Strengths and limitations

The main strengths of this study are the use of data from a large, heterogeneous sample of parents, both mothers and fathers, of preschoolers, and the use of advanced analysis methods, including CFA to validate the CEBQ, and SEM to test a theoretical model of child eating behaviors and their association with parental feeding practices and attitudes, controlling for child and parental characteristics. The CFQ was previously validated with CFA in a Swedish sample of preschoolers and proved reliable for the present population. It is also important to note that the study included both clinical and non-clinical populations, with a satisfactorily high response-rate (46.4%); this response rate was similar to the levels obtained in the previous Swedish validation study of the CEBQ [69], as well as in the Dutch [32] and the Australian validation studies [78]. The study’s main limitation is the self-reported nature of the measures of parental and child behaviors; although self-reporting is the most practical way to assess behaviors in a large-scale survey, we acknowledge that it may be subject to participants’ biases. Moreover, the cross-sectional design of this study does not allow us to determine the causality of the studied phenomena. It should be noted, however, that the questionnaires used in this study—the CEBQ, CFQ and LBC—are currently part of an assessment battery designed to evaluate the More and Less Study (ML) [75], an ongoing, comprehensive study of treatment interventions for preschoolers with obesity. ML will evaluate whether child eating behaviors and parental feeding practices are modifiable as a result of the intervention, and will also assess the relative importance of parental and child sociodemographic factors, thus advancing our ability to assess causality for child eating behaviors and parental feeding practices.

### Practical implications

Previous studies have found that excessive pressure to eat and restrictive feeding practices are counterproductive, with children eating less when being pressured and more (even in the absence of hunger) when being restricted [1, 39]. Our study found that pressure to eat is associated with parents’ perceptions that their children undereat, and that restrictive feeding is associated with parents’ perceptions that their children overeat, in the presence of parental concerns about children’s overweight. Thus, we suggest that parents could benefit from skills training and practical guidance on responding effectively to children’s eating behaviors. Indeed, group intervention programs for parents of children with obesity, which include training on how to respond to children’s eating behaviors, have shown promising results [66, 98]. We suggest, moreover, that preventive educational programs on how to guide children with a big or small appetite should be offered to all interested parents, regardless of the children’s weight status. These general educational programs could be offered in both clinical and non-clinical settings, particularly child health care centers and preschools, which can offer effective outreach to the majority of the target population and are easily accessible to parents of young children.

## Conclusions

This study demonstrated that the Swedish version of the CEBQ is valid and reliable in assessing dimensions of eating behaviors among preschoolers. Moreover, through examining associations between the CEBQ and the CFQ, and the CEBQ and the LBC, this study found that dimensions of preschoolers’ eating behaviors are associated with parental feeding practices and parental concern about child weight. Specifically, parental pressure to eat was strongly associated with child food avoidance and parental restrictive behavior towards children with big appetites was especially pronounced when parents were concerned about child weight status. We suggest, therefore, that obesity prevention and intervention programs targeting parents of preschoolers should take into account parental concern about child weight status and managing children’s eating behaviors, and promote positive, child-responsive approaches to feeding.

## Abbreviations

BMI: Body Mass Index
BMI SDS: Body Mass Index Standard Deviation Score
CFA: Confirmatory Factor Analysis
CFI: Comparative Fit Index
CFQ: Child Feeding Questionnaire
CONF: Confidence scale (scale of LBC)
DD: Desire to Drink (subscale of CEBQ)
EF: Enjoyment of Food (subscale of CEBQ)
EOE: Emotional Overeating (subscale of CEBQ)
EUE: Emotional Undereating (subscale of CEBQ)
FF: Food Fussiness (subscale of CEBQ)
LBC: Lifestyle Behavior Checklist
MLR: Maximum Likelihood with Robust standard errors estimation
RMSEA: Root Mean Square Error of Approximation
SD: Standard Deviation
SE: Slowness in Eating (subscale of CEBQ)
SEM: Structural Equation Modeling
SR: Satiety Responsiveness (subscale of CEBQ)
SRMR: Standardized Root Mean square Residual
TLI: Tucker-Lewis Index
